# Identification of the H&S (Health and Safety Factors) Involved in Infrastructure Projects in Developing Countries-A Sequential Mixed Method Approach of OLMT-Project

**DOI:** 10.3390/ijerph17020635

**Published:** 2020-01-19

**Authors:** Ahsan Nawaz, Xing Su, Qaiser Mohi Ud Din, Muhammad Irslan Khalid, Muhammad Bilal, Syyed Adnan Raheel Shah

**Affiliations:** 1 Institute of Construction Project Management, Collage of Civil Engineering & Architecture, Zhejiang University, Hangzhou 310058, China; ahsanklasra@zju.edu.cn or; 2 Department of Management Science, Bahria University Islamabad, Lahore Campus, Lahore 54600, Pakistan; 03-298181-041@student.bahria.edu.pk; 3 School of Economy & Management, Harbin Institute of Technology, Harbin 150001, China; qaiser@hit.edu.cn; 4 Department of Civil & Environmental Engineering, Hanyang University, Wangsimni, Seoul 04763, Korea; irslankhalid@hanyang.ac.kr; 5 Department of Civil Engineering, Pakistan Institute of Engineering & Technology, Multan 66000, Pakistan

**Keywords:** construction, health, safety, infrastructure projects, delphi survey, OLMT-project

## Abstract

Urbanization is playing a key role in big cities of developing countries, which, in effect, is increasing the population. This study takes care of the mega infrastructure project (Orange Line Metro Train (OLMT)) to explore and identify the H&S (Health and Safety) factors that affect the local residents and the main key stakeholders working on the project. A Sequential Mixed-Method approach of the OLMT-project includes qualitative and quantitative methods were adopted. The data have been collected from the targeted population working on the OLMT-project through a questionnaire. The main key finding of the study indicates that poor planning and a lack of communication between the public and government led to frustration. The most significant factors that identified in the study were unsafe to work practice, project scope constraints, lack in technical and material support, unsafe/bad condition, health/environment degradation, declination and loss of resources and time, no proper emergency system, and negligence in adopting safety rules and laws. The study also revealed that the consensus should also be noticed between the key stakeholders (e.g., contractors, clients, safety officials, academia) in the second round of the Delphi survey of the project. The study findings will help the key stakeholders to prioritize their energies towards attaining zero levels of inadequate health and safety practices in infrastructure projects. The study outcomes can also be generalized for the other developing countries having a similar work scenario.

## 1. Introduction

The migration of people from villages to cities is the concept of urbanization [1,2]. Urbanization is triggered due to change in local market conditions, poverty, unemployment, lack of basic facilities, and resources for growth as the push factors to move from villages, while all of the factors include technological advancement, better policies, better opportunities, employment, industry, and education [3,4,5]. The urban environment includes advancements in the shapes of the streets, its structure, nature, and the potential to foster the business economy of any country [5]. In any developed country, overall 80% of streets used to build and esteem the business activity for maintaining the urban environment [1,5]. This trend of the urban environment is observed throughout the world and it is at its peak in Japan, India, Bangladesh, Mexico, the USA, Brazil, Nigeria, Indonesia, and Pakistan [2,6,7,8]. The countries start to urbanize with the development of technology, advanced civic amenities, opportunities for education, facilities for transport, business, and social interaction [2,9]. In the process of the urbanization, the infrastructure of a country has great importance for its development and economy boosting [10]. Infrastructure-projects involves the complexities and safety issue that leads to an unsafe environment and their environmental impacts for different project key stakeholders and the residents [1,5]. Infrastructure-projects have continued to cultivate and commercialize the world economy and globalization has driven us to scientific advancement, convenient communication, rapid transportation, and facilitate the development of the construction process [4,11,12]. The Governments in developing countries, where approximately 85.4% of the world’s population lives, develop infrastructure projects to achieve their social and economic sustainable development objectives [13,14]. Infrastructure projects are prevailing in developing countries, and during the recent decades, many infrastructure projects are in their execution, development, and planning phases [6,7,15]. In South Asia, Pakistan is among the most rapidly urbanizing countries [9], and the part of the population in urban areas has increased from 17% to 37% from 1951 until 2010 and up to 43% to 2018 survey [12,16,17,18]. In addition, it is calculated that half of the population will live in urban areas in the next 10 to 15 years [18,19]. It is estimated in recent studies that the planet will be populated up to 2030 of 8.5 billion people [20]. The constant trends for the urbanization will have an enormous impact on the climate change and the environmental destruction. Furthermore, this will produce a unfavorable environment for the urban habitants while maintaining the developing cities [1]. According to the statement of the Saldaña-Márquez, 2019 half of the places to be urbanized up to the 2023 has not been built yet, Hence, there is an esteemed need to establish the environmental codes and practices in developing countries to counter the environmental destruction for the human beings [1,5]. Population growth and migration are the major forces behind urban growth [7,19]. Migration in developing cities is major due to the search for employment, education, and better opportunities [15]. An increase in population demands more public transportation [21]. The environment might get impacted by the infrastructure-projects and affect the surroundings of residents; thus, it is not rare to attract criticism from several stakeholder groups. The aim of any public infrastructure and construction (PIC) projects is to improve the comfort of society [22,23]. The development of any public infrastructure construction (PIC) project from the initialization phase to the handover phase can have controversies, and it might affect the interests positively as well as negatively. Representatives of these interests are referred to as the project stakeholder [2,24]. OLMT (Orange Line Metro Train) is one of its kind projects, and yet no study has taken place regarding the health and safety of residents, or issues arising due to such a project. Therefore, the literature on similar projects from the rest of the world is used to support the research. This study has a case scenario, which presents to identify and explore the contributory factors that cause an unsafe environment in the construction industry.

Society contribution and consensus are important in the planning of mega construction projects [25]. A systematic participatory framework for PIC projects was proposed in the “express rail link project” from the findings and lessons learned of the China–Hong Kong express rail service linkage project. The researchers focused on the methodology of consensus building and keeping because of previous practices, suggested for improvements in it. In consensus building, they suggested that more effort was to be made in the growth of saturation point reaching of all the stakeholders. From the study, it was proven that, for the success of the project, all of the stakeholders are important. They also concluded that stakeholders were also affected by the project. Thus, their consensus is very important and should be considered while the planning phase, so that risks and people issues could be mitigated [25].

Previous research studies also prove that infrastructure-projects can often be planning disasters and they can generate heavy impacts on society as well as cost over runs [26,27]. It is due to the result of a lack of public participation [28]. The benefits from such projects occur on a certain level. Municipal area level gets benefited, residents may get adversely affected, and may incur a lot of issues [29]. It may include mobilization and displacement due to space formation for new facilities, increased traffic, noise, and air pollution. In the world, Korea became the fifth country to operate and own a high-speed railway in 2004, called ‘Korea Train Express (KTX)’. Mega construction projects involve a lot of planning and management for their success. If planning is insufficient, the project leads to delay or may fail, as the same case with the KTX project. Five major delays were found in the KTX project, which is “Lack of owner’s abilities and strategies to manage hi-tech oriented mega-project, frequent route changing triggered due to conflicts between public agencies and growing public resistance from environmental concerns, inappropriate project delivery system, lack of proper scheduling tool tailored for a linear mega project, and redesign and change orders of main structures and tunnels for high-speed railway” [30]. There are a variety of procedures and programs that can be planned that support the performance of safety and to avoid environmental hazards for project phases for employees as well as for the public [1,2,5].

Safety practices in construction companies in developing countries are not so developed and have proved to be risky due to the involvement of different types of project complexities [2,31,32]. According to one’s findings, in the construction industry of developing countries (i.e., India, Bahrain, Bangladesh, Nepal, Pakistan, Iran, Malaysia), safety performance is not satisfactory, which are creating the environmental risk, in addition, stakeholders only emphasize reducing time and cost [21,31,33]. The client does not allocate any safety budget and according to results, 61.8% of construction companies do not have any policy related to safety or workers as well as locals stakeholders [8,15,34]. Construction safety is considered as a second responsibility in the construction projects [18,35,36], which causes an unsafe environment and makes project risky [12,32,37,38].

The safety policies are almost invisible in the construction industries of developing countries; furthermore, the occurrence of the work-site accidents is increasing day by day [31,38,39] due to the low level of the inspection and regulating bodies besides [40,41], in addition work-site accidents are threatening the work environment and construction safety [41]. The main cause of the work-site accidents is low level of workers trainings [35,37], and this is considered to be the most ignored safety practices in the construction industry [34,42,43]. Safety training programs are also not conducted [36,37], due to which workers do not know about their job-specific safety before start of their project work [15,32], though from the review, it shows a direction towards the results that more importance was given to measuring the factors of projects delays factors, project planning and scheduling, and causes of accidents [40,44], a little attention was given to identifying the major factors that produce an unsafe environment and environmental codes for the residents and the key stakeholders of the infrastructure projects in developing countries.

The main objectives of the Study is: (i) to study, identify, and explore the H&S (health and safety) factors, (ii) to prioritize the H&S factors in term of their significance, (iii) to apply the practical knowledge of construction health and safety in infrastructure projects, and (iv) to establish the guidelines for the construction enterprisers regarding H&S and environmental impacts for the future. According to author knowledge, no study has yet been investigated to prioritize the fundamental roots of unsafe construction environment and environmental impacts in infrastructure projects of Pakistan. The study findings will also provide the path to the key stakeholders (i.e., contractors/subcontractors, clients, consultants, academia) to prioritize their efforts towards achieving an effective and safe environment for the construction projects.

## 2. Research Methodology

**Figure 1:**
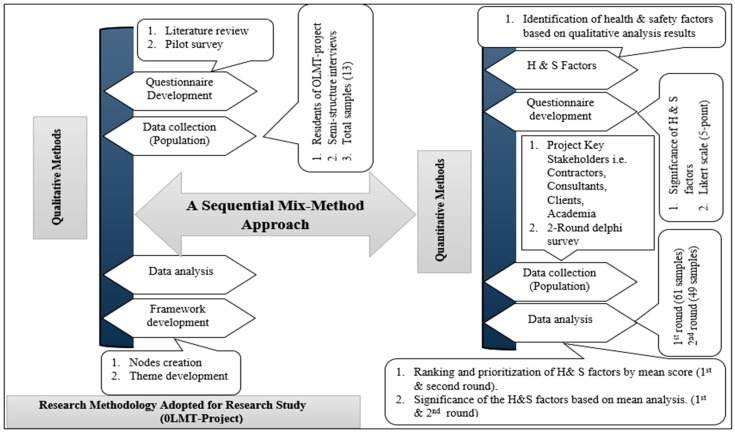
Research Methodology adopted for (OLMT) study.

This section presents the research design involving both Narrative (A type of Qualitative methods) and Quantitative methods (i.e., Delphi survey) to overcome the limitation of the single design [4,15,45]. The data (i.e., Semi-structured interviews) were collected from the targeted population (local residents of the OLMT-project) by adopting the Narrative technique. A total of 32 nodes were extracted after the screening of the raw data while using the qualitative analysis. Later on, these collected nodes were trailed by the qualitative methods (two-round Delphi Survey) from the key project stakeholders (targeted population) to measure and prioritize the identified factors (themes) as their significance level. The methodology is illustrated in detail in the below segment (as shown in Fig.1).

There are two types (i.e., Qualitative & Quantitative) of data that have been collected in this study and, furthermore, it can be described in two ways. (i) semi-structured interviews (narrative technique), which were taken from the local residents (targeted population for qualitative methods) living around the “OLMT-project”. In this method, data have been recorded in audio-tapes and later on it was screened out and manuscript to generate the nodes (semi H&S factors) by the help of statistical tools (NVIVO vs 10, Statisticians). In addition, expert opinion was also a major part of this step. A total of 10 major themes (H&S factors) were selected from the selected semi factors (nodes). (ii) The identified themes were used again for quantitative analysis (two-round Delphi survey); however, for the Delphi-Survey, a questionnaire has been developed based on the (five-point Likert scale) to collect the data from the target population (project key stakeholders i.e., contractors, safety officials, client, academia). The instrument (questionnaire) that was adopted for the semi-structured interviews consisted of 15 major open-ended questions. The construction industry experts validated these open-ended questions using the pilot survey. The questionnaire (instrument) used for the narrative technique (a type of qualitative study) was designed in such a way that it can be used to collect the data from the targeted population easily [8,15]. Interviews were conducted to include views of the people who lacked education and were unable to answer the questionnaire in writing. More questions were to be asked at the spot and a complete interview was to be audio recorded for complete narration and transcription in original form. There was a total of 26 stations, and thirteen areas were spotted (each area comprises two stations) for the interviews to complete the process of a qualitative study. The 26 stations and the thirteen areas of the “OLMT-project” are depicted below in Table 1. A Delphi survey was conducted based on narrative study (semi-structured interviews), which consist of (i) demographics (i.e., stakeholder group, experience, a position at project sector) and (ii) H&S factors (identified themes). The targeted population (key stakeholders i.e., contractors, clients, academia, safety officials) were asked to prioritize the H&S factors according to their significance based on Likert scale (five-point) by using the Delphi technique, with 1 showing “not important” 2 “somewhat important” 3 “important, 4 “very important”, and 5 “extremely important” [4,15,46,47,48]. The success of the Delphi method based on the experience of the panel members [2,4,47]. According to Ameyaw and Chan (2015), the guarantee of the reliability of the collected data depends upon the respondent’s organization and its versatile experience regarding its particular working domain (sector) [49]. Hereafter, the panel would be a mixed-group enriched with diverse experience [50]. The panel size is suggested by Hon et al. (2010) to be seven members that can be amplified up to 50 [50,51]. An effort has been made for this study to collect the minimum 10 responses from each respondent (e.g., contractors/subcontractors/client, safety officials, academia), with each member having a diverse experience. By adopting this selection method, it was felt that the results achieved would be impartial and more reliable.

The objective of the Delphi survey is to gain a specific opinion of the different groups having versatile experience in a particular area [27]. A criterion has been set for the population (project key stakeholders), who must have a registration from the Engineering Council (PEC) as a professional member i.e., professional engineer, client, and contractor. A Delphi-survey is commonly used in the construction management field [4,52]. A two-round Delphi survey was adopted to verify as an accurate and precise technique for element prioritization [22,43,48]. The complete research methodology adopted for (OLMT) study is illustrated in detail (as shown in Figure 1).

In the first phase, 61 responses were collected. Table 2 shows the distribution of the respondents along with their versatile experience. The data were collected in the first round through field links, postal mails (printed instruments), and the face to face meetings. The identities of the respondents were concealed to each other to get the more reliable results [53], and it continues to the repetition of the round until the consensus was established and sharing the desired statistical results to the responders after the completion of the first round. The data that were collected from the first round were used for the statistical analysis to test whether the consensus had been made between the respondent groups or not. The mean score was calculated for the second round and presented to the respondents using the online questionnaire. It was an open choice that was given to the respondents to either sustain or change their original grading. Nevertheless, for this round, the respondents lessened up to 49, as shown in (Table 2) with their distribution as clients (11), academia (11), safety officials (13), and contractors (14). The Delphi survey was stopped when the consensus was made between the respondent’s group. The results of the data analysis are presented in the next section.

## 3. Materials and Methods

This section signifies two types of analysis. (i) qualitative analysis (i.e., creation of nodes, themes/factors development, intercorrelation of the H&S factors, (ii) quantitative analysis (i.e., normality & reliability of the data, ranking, and prioritization of H&S factors based on mean scoring.

### 3.1. Qualitative Method

Table 3 shows the 32 nodes and their occurrence on the different stations of the OLMT-project. There is an increase of population told by the interviewee of area 1,3,8,11 and no public transport facility was provided (2,4,6–10) from the last six years, which take us to the situation that there was also poor condition of the roads (1,3,4,6,8,9,10,11) and unsafe traffic route (4,5,7,9–12) and declination in public daily routine (2,4,6,8,10,11,13) through the execution of the project. The condition becomes worst when rain (1–4,6,8–12) happens due to God’s will situation [17,32]. The interviewee of all areas elaborated that there is an increase in population due to which current services did not fulfill the transport requirements of people, which means there was a lack of public transport [54] and lack of public transport results in an increase of private transport [22,55].

According to the study of Qureshi (2010), there was a poor condition and lack of public transport, as well as handy car loans via banks, causes a lot of increase of private transport in Lahore [55]. This drove us to a major issue of traffic congestion [7,56] due to which people suffer a lot. The interviewee of AREA 1,4,8, and 11 said that there was no need for the OLMT-project route in their areas, but the interview of AREA 2,3,5,6,7,9,10,12, and 13 said that they were optimistic regarding the future benefit that this will facilitate in travelling and there was a need of public transport in this area. Zaman (2012) clarifies, in his study, that there was an increase in transport on roads in urban areas of Malaysia which was causing severe traffic congestion, air pollution, and deteriorating the environment of cities [57]; the same situation has accrued within Lahore [54]. Most of these nodes were common in all areas of the OLMT-project. However, few areas gave differently results as according to the interviewee of AREA 1,2,4,7,8, and 10, there was a proper use of sign-boards, but, according to the interviewee of AREA 3,5,6,9,11–13, there was no proper use of sign-boards. This shows that proper usage of sign-boards is missing in seven out of thirteen areas. According to the interviewee of AREA 1,3,4,6–8,10,11, and 13, they were not informed regarding the start of OLMT-project in those areas, but according to the interviewee of AREA 2,7,5,9 and 12, they were told regarding the start of the OLMT-project in those areas. This clearly shows that residents of many areas were not informed about the project before its start, which drives us to the lack of project planning [7,31,32,56,58]. Wang & Zuo (2016) describe in his study that the low level of information leads to limited knowledge, limited knowledge, then consequence leads to irrational behavior by the public [44], which justifies the results of our study.

The Interviewee of AREA 2,4,5,7,9,10–13 said that there were no piles of material or mud in those areas, but the interviewee of AREA 1,3,6, and 8 said that piles of mud and material were spread [9] due to the construction of OLMT-project, which clearly shows that there were ten areas in which piles of material and mud were on pavements due to which unsafe conditions occurred in these areas [59]. According to Wang & Zuo (2016), during the project development, the social and environmental effects of projects have been misjudged and have not usually been taken into account [44]. The same case was with the high-speed rail mega-project in Germany, which was condemned for not taking environmental disorders into account [28]. These issues disrupt communities and habitants [4,9,55,60].

The interviewees also noticed the use of the heavy machinery (1,2,3,6,7,9,11) causing so much noise pollution [2,19] and no advancement in tools [6,61] on site (1,2,4,5,6,9,10,11). It was also revealed by the interviewee that there was debris (1,3,4,6–8,11) everywhere and environmental hazards (2,4,6,9–11) were also noticed on the sites. In addition, people also have a health problem [2,62] i.e., drug addiction (3,5,6–9,11–13).

According to interviewee of AREA 1,2,4,6, and 13, workers of these areas properly use personal protective equipment, but, according to the interviewee of AREA 3,5,7–12, the workers of these areas use partially or no personal protective equipment, which shows that safety practices were not so good in all areas, which justified the findings of the Khan (2012) [15]. According to his study, the most ignored safety practices in construction industry are related to health and safety training [12,15,22], which is not provided to employees/ workers of subcontractor, due to which they are not aware of safety practices and it results in causing a hazardous situation for themselves and others around, which cause incidents [17]. Safety training programs [2,31] are also not conducted, due to which technical workers do not know about their job-specific safety before the start of their project work. This leads to unsafe work practices and conditions. [4,43,63]. According to interviewee of AREA 1,2,4,6–8,10–12, their businesses were in loss with the start of the OLMT-project, which shows that there was a loss of resources [15] due to the construction of OLMT-project in nine areas out of thirteen. There was traffic congestion [64] in the area of 2,4, and 6, and the traffic condition was normal in other areas told by the interviewees.

The interviewee of AREA 2,4,6,8–11 revealed that there were a lot of work accidents [15] and the death of locals and workers (2,4,6,8,10) in these areas. The interviewee of AREA 8 has revealed a death case, that there was the death of 18–20 workers of the OLMT-project due to falling of the wall. Similarly, according to the interviewee of AREA 6, there was the death of seven people due to the accident of crane, which is on work of OLMT-project, with a rickshaw having seven passengers, one of them was a boy of eight years. The Interviewee of AREA 5 mentioned a car accident in which the back screen of the car shattered into pieces due to the fall of tools during cleaning pillars from the worker of the OLMT-project team. Similarly, no emergency system was installed [15] for accidents (1,3,4,7,9,10,12,13) and no insurance policy for health of workers (1,2,3,7,9,10,12,13). Unskilled workers (1,4,6,8,11,12) and no awareness for the project execution (3,5,8,10,11,13) were also identified from the interviewees [9,56]. The outsourcing of materials (3,4,6,8,9,10,12) was at its peak and there was not a proper reporting system between teams and workers (2,5,6,7,11,12), which shows a complete lack of planning and work quality [7,32,65].

#### Framework Development of OLMT-Project for Narrative Study

From these all major nodes (semi factors), which were the most common in all thirteen interviews, ten themes (H&S factors) were generated. They were developed after finding nodes. These 10 major themes/factors are as follows:

(1) Poor Project Planning [15,66]
(2) Limited Information [7,18,31]
(3) Unsafe work Practices [9,17,67]
(4) Project Scope Constraints (Schedule, Budget) [7,65]
(5) Lack of Technical & Material Support [68,69]
(6) Unsafe/Bad Condition [40,70]
(7) Health/Environment Degradation [12,15]
(8) Declination and loss of Resources & Time [19,31,67]
(9) No Proper Emergency System [7,19]
(10) Negligence in Adopting Safety Rules and Law [17,31,56,58,67]

**Figure 2:**
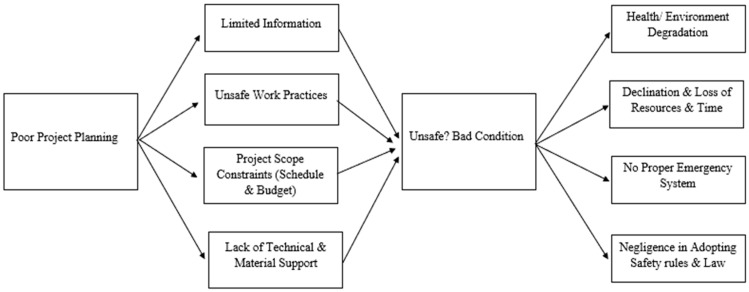
The Developed framework of health & safety factors of (OLMT-project).

Developing the framework is the final step in the interpretation of data in the qualitative approach [7,31,43], which is shown as below in Figure 2, that all of the themes correlate with each other [8,15], which will be explained in the next section i.e., data analysis.

According to the developed framework, with the increase in population [2], available transport facilities did not fulfill the transport requirements of people i.e., lack of public transport [31]. The local government started the OLMT-project in order to compensate for this issue [71]. However, at the start of the project, due to poor planning [43] and keeping them uninformed and not considering issues of stakeholder resulted in limited information, lack of awareness, and scope creep [56]. Support contractors and sub-contractors did not follow the safety rules and practices due to poor planning and lack of technical knowledge and material [64], which triggers unsafe work practices [54]. Limited information and unsafe work practices lead to the poor condition of roads, hazardous situations, piles of material, and combinedly prompt unsafe/poor conditions [15,65]. Air pollution, dust, noise, and many other associated risks, including health risks, like breathing problems, allergies, and cough, etc. increased a great deal and affected the people adversely due to the construction of OLMT-project [9,15,31]. This triggered two major themes i.e., health/environment degradation and decline and loss of resources [17]. A lot of people have been affected due to the unsatisfactory conditions of environment and health. Resources, like buildings, have also been affected and human resource [34] has been affected a great deal. The passerby’s also face similar problems, as those people are also locals of that area for a certain period of time. A lot of accidents included injuries and deaths of common people, which involved both the passersby and the residents, which triggered two other two major themes/factors i.e., no proper emergency system and negligence in adopting the safety rules and laws [6,7,31,56].

### 3.2. Quantitative Method

#### 3.2.1. Normality & Reliability Test

A statistical tool that was developed by the IMB called Statistical Package for Social Science (SPSS vs. 25) and Microsoft Excel was used to collect and analyze the data. Shapiro–Wilk-normality test was performed to check either data was normally distributed or not. The results show that the identified significance value was less than 0.05 (*p* < 0.05) for both rounds of the Delphi survey of all the factors. In this case, it requires the non-parametric test for further analysis [4,15]. Cronbach’s coefficient α was conducted for both rounds of the Delphi survey, besides, the statistical test was led for each respondent group to access the reliability of the collected data. The results show that all of the values were more than the 0.7, as shown in (Table 4 and Table 5), and the data are reliable and can be conceived for the more analysis [15,32,72].

In this table, factors represents as e.g., A = Poor project planning; B = Limited information; C = Unsafe work practices; D = Project scope constraints (schedule, budget); E = Lack in technical & material support; F = Unsafe/bad condition; G = Health/environment degradation; H = Declination and loss of resources & time; I = No proper emergency system; and, J = Negligence in adopting safety rules and law.

#### 3.2.2. Ranking Based on the Mean Score

The health and safety factors were calculated and organized from not important to extremely important based on their mean, in addition, the respondent’s groups were asked to identify and prioritize the H&S factors on the scale of 1 to 5, where 1 represented the not important and 5 represented extremely important. Overall, 10 factors were ranked based on their mean score not only for all respondents, but also for each respondent group (Table 4 and Table 5). Table 4 shows the ranking of the H&S factors of the first round. The factor “Limited information” (mean 4.244) was selected as the very important used by the respondents and ranked 1st based on their mean. Likewise, the factor “Poor project planning” was selected as the important (mean 3.246), as per their significance, and then ranked 10th in the group. The “factor” Unsafe work practice (mean 4.372) was ranked first (Table 5) by the respondents of the second round of Delphi survey and conceived as very important, as per their significance from the group. Similarly, the “factor” Negligence in adopting safety rules and the law was ranked 10th (mean 3.372) by the respondents and considered as the very important as per their unified significance level. The deep analysis of both rounds of the Delphi survey revealed that some of the respondents have refined the ranking of the factors in the second round and some have switched their ranking in second rounds. The factor A (mean 3.246), C (mean 3.836), D (mean 4.000), E (mean 4.033), F (mean 3.787), and H (mean 3.475) enhanced their ranking in second round; A (mean 3.629), C (mean 4.372), D (4.030), E (mean 4.327), F (mean 3.794), and H (4.088) from ranked (10th,6th,5th,4th,7th,8th) to (8th,1st,4th,2nd,6th, 3rd). In the same way, the factors; B (mean 4.244), G (mean 4.230), I (mean 4.049), and J (mean 3.262) has substituted their ranking in second round; B (3.749), G (mean 3.571), I (mean 3.992), and J (mean 3.372) from (1st,2nd,3rd,9th) to (7th,3rd,5th, 10th). The consensus between the respondent’s groups (e.g., contractors, clients, safety officials, academia) was also developed after the second round of the Delphi survey. In the first-round, contractors and academia had a consensus for factor (A and B), while in the second round the consensus exists between clients and academia (ranked fifth) for factor A (Table 4 and Table 5). Similarly, in the first round, the factor D (ranked 3rd) has consensus between clients and safety officials and, in the second-round, consensus exists between clients, contractors, and safety officials for factor D (ranked 4th).

In this table, factors are represented as e.g., A = Poor project planning; B = Limited information; C = Unsafe work practices; D = Project scope constraints (schedule, budget); E = Lack in technical & material support; F = Unsafe/bad condition; G = Health/environment degradation; H = Declination and loss of resources & time; I = No proper emergency system; and, J = Negligence in adopting safety rules and law.

The Factors E and F (ranked 4th,6th) have consensus between clients and academia in the first-round, while in the second-round factor G (ranked 3rd) has consensus on clients and contractors. The factors H and I (ranked 10th,9th) had consensus between (contractors, safety official) and (safety official and academia) in the first round. Though the three respondent groups (e.g., contractors, safety officials, academia) have consensus for factor H (ranked 3rd) in the second round of the Delphi survey. The factor J (ranked 9th) has consensus between the contractors, safety officials, academia in the first round. However, the consensus has also been noticed between (clients, safety officials) and (contractor and academia) for factor (ranked 6th and 9th) in the second round. All of the H&S (health and safety) factors were ranked based on their mean score gained after the second round of the Delphi survey. The scale has been drawn to measure the significance of each factor as: not important (mean <1.5); somewhat important (mean 1.51 ≤ mean ≤ 2.5); important (mean 2.51 ≤ mean ≤ 3.5); very important (mean 3.51 ≤ mean4.5); and, extremely important (mean ≥ 4.5) [15,63,73].

The results in (Table 6) indicated that the mean score of all the factors in both rounds were less than the mean score 4.5 (extremely important), which shows that their significance level was very important (3.51 ≤ mean score ≤ 4.5) in both rounds which is quite a higher level of significance [73].

The results also direct that the two factors “poor project planning” and “declination and loss of resources & time” has increased their significance level based on their mean score from “important” to “very important” (highlighted by upward arrow) [74,75,76], other factors have a significance level based on mean score as “limited information (significance as V. imp) in both rounds [15,20,77,78,79], unsafe work practices (significance as V.imp) in both rounds [61,76,80], project scope constraints e.g., schedule, budget (significance as V.imp) in both rounds [81,82], lack in technical and material support (significance V.imp) in both rounds, unsafe/bad condition (significance V.imp) in both rounds, no proper emergency system (significance V.imp), negligence in adopting safety rules and laws (significance Imp) in both rounds of the Delphi survey [15,20,49,77]. Likewise, the significance level of the factor “health/environmental degradation varies from the first-round (V.imp) to second round (Imp) and the factor “declination and loss of resources & time) from important(first round) to very important(second round) [15,19,79,82]. This study witness to mark that not a single factor was graded below 2.5 and each factor has a significant level as important or very important. Hence, it is concluded that all of the identified factors have a significant role in the infrastructure project e.g., OLMT-project with a perspective of health and safety [4,15,83].

## 4. Discussion

The study has presented the H&S factors that cause the construction process of infrastructure projects to slow down and produce a risky environment [4,34] for the workers. The study based on the mixed-method strategy of OLMT-project encompassed semi-structured interviews and the Delphi survey. The qualitative data were collected from the targeted population (local residents of the OLMT-project) while using the semi-structured interviews (occupying Narrative technique). A total of 32 nodes were generated from the qualitative analysis, which was lagged by a two-round Delphi survey (quantitative method) from the targeted population (key stakeholders i.e., contractors, academia, client and safety officials) to prioritize the identified factors as per their significance. The qualitative data were screened out and went for analysis by the help of statistical tools (NVIVO and Statisticians), which results in the extraction of the total (10) Health and Safety factors. The Delphi survey (quantitative analysis) was conducted to check the consensus between the respondent groups of clients, contractors, academia, and safety officials, which resulted in a rational consensus that was noticed between the key stakeholders of the project, as shown in Table 4 and Table 5. For further analysis of the data, Cronbach’s α was applied to check the reliability [17,32] of the data, which gives higher values in both rounds of the Delphi-survey.

The Health and Safety Factors (H&S) were signified and prioritized through mean score ranking analysis. All of the H&S factors are concise in the table (6) in order of their significance, which is from “extremely important” to “important”, indicating that there is an extreme need to emphasize on the all prioritized health and safety factors of the construction projects [4,15].

- The analysis shows that the most significant factors e.g., poor project planning; Declination and loss of resources and time; limited information; No proper emergency system; unsafe/bad condition; project scope constraints (schedule, budget) and lack in technical and material support were graded as very important [8,9,15] and the factor (negligence in adopting safety rules and law) graded as “important” in the second round of the Delphi-survey, as indicated in Table 6.
- The respondent groups highlighted that these major factors have a strong consensus, which was also noticed, besides the academia and the clients group having strong consensus on the factors “poor project planning” after the second round of the Delphi survey [15].
- A strong consensus has been developed between the client, contractors, and safety officials for the factor “project scope constraints (schedule, budget)” after the second round of Delphi survey which shows that it is the most significant factors as per their occurrence and significance level [4,17,43]. The factor (declination and loss of resources and time) was graded as “most important” and all of the respondent groups were agreed on their common consensus [4,56]. The factor “unsafe work practices” emerged to be the first most significant factor [34] and the factor “health/environment degradation” was graded as the 10th most significant factor in this study [4,15,84].
- Although the significance level of the next eight factors ranked from second to ninth in (Table 6) were measured as very important (poor project planning, limited information, unsafe work practices, lack in technical & material support, project scope constraints (schedule, budget), unsafe/bad condition, declination and loss of resources and time, no proper emergency system) to important (health/environment degradation, negligence in adopting safety rules and law) [4,35].

## 5. Conclusions 

This study presents the health and safety (H&S) factors involved in the process of construction in infrastructure projects, which permits the identification process of the health and safety issues in construction projects among all sectors. Though the non-availability of reliable data for these types of complex infrastructure projects narrowed this research study to evaluate and identify the primary factors that cause health and safety issues in infrastructure projects in developing countries. Overall, 32 nodes (semi factors) were generated by using the narrative technique (semi-structured interviews) from the targeted population (local residents); furthermore, these identified nodes were rectified and then screened out to extract the themes (major factors). A Delphi-technique (quantitative analysis) was applied to test the consensus between the key stakeholders (e.g., contractors, clients, academia, safety officials). The major outcomes taken from the study after applying the research methods are as follows:

- There was a positive consensus achieved between the respondent groups after the second round of the Delphi-survey and this consensus was prioritized for significance level. The consensus that was perceived between the client of having mean (3.930) and contractor (mean 4.177) for the factor (poor project planning), which was ranked fifth after the second round of the Delphi survey, which authenticates the findings of the [4,9,17]. A mutual consensus was also noticed between all of the respondent groups for the factor “declination and loss of resources and time” after the second round.
- The factors “unsafe work practice” having an accumulative mean value of (4.372), lack in technical and material support (mean 4.327) was ranked as (1st and 2nd) with a significance level of (very important). Besides, the factor “Negligence in adopting safety rules and law” with the mean value of (3.372) and the factor (Health/environment degradation) with the mean value of (3.571) was graded as “important” and ranked as (10th and 9th).
- All of the respondent groups were agreed except the academia for the factor “Project scope constraints (schedule, budget)” with the mean value of (4.260, 4.211, 4.225) and having a priority of (ranked 4th).

There are many stakeholders involved in their own responsibilities in construction projects. The contractors and the subcontractors are considered to be the major’s stakeholders in the construction sector, because they are answerable for the project budget, execution and project timeline limit [31,63,85]. In addition, all of the budget and the project scope is based on the clients and contractors [31] and the project safety depends upon the safety officials [9]. Hence, these stakeholders need to play a vital role to achieve better safety performance in the construction industry, besides, they have also been suggested to regulate the adequate safety practices, training, and safety awareness campaigns to enhance the safety compliances.

This study is limited to only infrastructure projects e.g., OLMT-project that helps to boost the economy of the country [31,41]. The stakeholders that are involved in the survey were limited to the enlisted firms under Engineering Council (PEC), Pakistan; furthermore, future research can be conducted in developed and non-developed countries to investigate the health and safety factors involved in the infrastructure projects. There is a major need to adopt and implicate the systematic approach of the health and safety practices in the local construction environment. Finally, the health and safety standards (codes) need to be developed for mega infrastructure projects in developing countries i.e., Pakistan.

## Figures and Tables

**Table 1:** Orange Line Metro Train (OLMT) areas and stations.

Station	Area	Station	Area
Ali Town	Area 1	Chaburji	Area 7
Thokar Niaz Baig		Lake Road	
Canal View	Area 2	GPO	Area 8
Hanjarwal		Lakshmi	
Wahdat Road	Area 3	Railway station	Area 9
Awan Town		Sultanpura	
Sabzazar	Area 4	UET	Area 10
Shahnoor		Baghbanpura	
Salahudin Road	Area 5	Shalimar Garden	Area 11
Bind Road		Pakistan Mint	
Samnabad	Area 6	Mahmood Booti	Area 12
Gulshan-e-Ravi		Islam Park	
Salamat Pura	Area 13		
Dera Gujran			

**Table 2:** Group Wise distribution of the respondents for two rounds of Delphi survey.

Distribution of Respondents	Working Experience of the Respondent’s Clients	Contractors	Safety Officials	Academia
3 year	3(4)	2(2)	6(5)	4(5)
5–10 year	2(1)	4(3)	1(2)	4(2)
10–15 year	5(4)	6(5)	5(3)	2(2)
>15 year	4(2)	6(4)	4(3)	3(2)
Total (61 for first and 49 for the second round)	14(11)	18(14)	16(13)	13(11)

Note: Digits in brackets “()” shows the respondents of the second round in Delphi survey.

**Table 3:** Health and safety nodes (semi factors).

Health and Safety Nodes (Semi Factors)	Area												
	1	2	3	4	5	6	7	8	9	10	11	12	13
Need for public transport	√			√							√		
Traffic congestion		√		√		√							
Increase in population	√		√					√			√		
No emergency system for the health of the workers	√		√	√			√		√	√		√	√
Lack of awareness for the execution of a project			√		√			√		√	√		√
Unskilled workers	√			√		√		√			√	√	
Pollution (environmental hazards)		√		√		√			√	√	√		
Lack of public transport provided		√		√		√		√	√	√			√
Not informed of the project OLMT to the local residents	√		√	√		√				√	√		√
No precaution measures (proper use of signboards)	√	√		√			√	√		√			
The need for technical knowledge				√	√			√		√	√		√
Untrained heavy machinery operators		√		√		√	√		√		√		√
Negligence in following laws against public and use of resources	√	√		√			√		√		√	√	√
Piles of mud (unhealthy environment)	√		√		√			√					
Health problems of the workers (drug addicts)			√		√	√	√	√	√		√	√	√
Fatal accidents (no emergency system installed)		√		√	√		√			√		√	
Use of heavy machinery (a sound issue at day & night)	√	√		√		√	√		√		√		
Govt negligence (no notice by any government authority for project execution		√		√	√		√	√	√		√	√	
Hazardous situations	√			√		√		√		√	√	√	√
No alternate routes (declination in people daily routine)		√		√		√		√		√	√		√
Loss in business	√	√		√		√		√	√	√	√	√	
Negligence in regulatory authority (lack of implementation of health and safety practices)			√		√		√	√		√	√	√	
Poor condition of roads	√		√	√		√		√	√	√	√		
Work-based accidents (exchange of workers)		√		√		√		√	√		√	√	
Debris	√		√	√		√	√	√			√		
Negligence of project team (no safety inspection against teams by the authorities)		√		√	√		√		√		√	√	
Worst conditions if rain happened (poor planning against god will situation)	√	√	√	√		√		√	√	√	√	√	
Lack of safe transport (route issues)				√	√		√		√	√	√	√	
Death of people		√		√		√		√		√			
Communication gap (no reporting system between teams & workers)		√			√	√	√				√	√	
Huge level of outsourcing which lacks in quality of work			√	√		√		√	√	√		√	
No advance tools & machinery to mitigate the time & budget constraints	√	√		√	√	√			√	√	√		

**Table 4:** First Round of Delphi Survey.

	All Groups		Clients		Contractors		Safety Officials		Academia	
Factors	M	R	M	R	M	R	M	R	M	R
A	3.246	10	3.100	9	3.067	8	3.588	8	3.177	10
B	4.244	1	4.000	3	4.267	1	4.725	1	4.515	2
C	3.836	6	3.714	5	3.733	6	4.225	4	3.615	8
D	4.000	5	4.000	3	4.211	2	4.250	3	3.900	7
E	4.033	4	3.929	4	3.678	7	4.088	6	4.308	4
F	3.787	7	3.571	6	3.767	5	3.838	7	4.000	6
G	4.230	2	4.143	1	3.822	4	4.588	2	4.362	3
H	3.475	8	3.500	7	2.944	10	3.063	10	4.577	1
I	4.049	3	4.029	2	3.844	3	4.188	5	4.154	5
J	3.262	9	3.329	8	2.989	9	3.350	9	3.515	9
Samples	61		14		18		16		13	
Cronbach’s α	0.824		0.821		0.842		0.782		0.892	

Note: M = Mean; R = Rank. 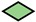 Present consensus between contractors and safety officials. 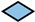 Presents consensus between clients and safety officials. 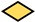 Presents consensus between clients and academia. 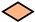 Presents consensus between safety officials and academia. 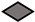 Presents consensus between contractors, safety officials, and academia.

**Table 5:** Second Round of Delphi Survey.

	All Groups		Clients		Contractors		Safety Officials		Academia	
Factors	M	R	M	R	M	R	M	R	M	R
A	3.629	8	3.930	5	3.067	8	3.345	7	4.177	5
B	3.749	7	4.460	2	3.267	7	3.725	5	3.545	6
C	4.372	1	3.674	8	4.745	2	4.455	3	4.615	1
D	4.030	4	4.260	4	4.211	4	4.250	4	3.400	7
E	4.327	2	4.689	1	3.656	6	4.658	2	4.308	3
F	3.794	6	3.271	9	3.767	5	4.838	1	3.300	8
G	**3.571**	9	4.557	3	4.456	3	3.278	8	4.362	2
H	4.088	3	2.345	10	2.644	10	3.063	10	3.257	10
I	3.992	5	3.679	7	4.867	1	3.268	9	4.154	4
J	**3.372**	**10**	3.739	6	2.925	9	3.560	6	3.265	9
Samples	49		12		14		13		11	
Cronbach’s α	0.834		0.721		0.742		0.882		0.892	

Note: M = Mean; R = Rank. 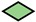 Present consensus between client and academia. 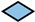 Presents consensus between clients, contractors and safety officials. 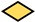 Presents consensus between clients and contractor. 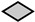 Presents consensus between clients, contractor, safety officials and academia. 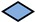 Presents consensus between clients and safety officials. 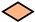 Presents consensus between contractors, safety officials, and academia.

**Table 6:** The significance level of health and safety factors.

H & S factors	First-Round			Second-Round		
	M	R	S	M	R	S
Poor project planning	3.246	10	imp	3.629	8	↑V. imp
Limited information	4.244	1	V. imp	3.749	7	V. imp
Unsafe work practices	3.836	6	V. imp	4.372	1	V. imp
Project scope constraints (schedule, budget)	4.000	5	V. imp	4.030	4	V. imp
Lack of technical & material support	4.033	4	V. imp	4.327	2	V. imp
Unsafe/bad condition	3.787	7	V. imp	3.794	6	V. imp
Health/environment degradation	4.230	2	V. imp	3.571	9	imp
Declination and loss of resources & time	3.475	8	imp	4.088	3	↑V. imp
No proper emergency system	4.049	3	V. imp	3.992	5	V. imp
Negligence in adopting safety rules and law	3.262	9	Imp	3.372	10	imp

Note: M = Mean; R = Rank; S = Significance and V. imp, very important; ↑ shows an increase in the significance of the factors from first round to second round.
